# Gender Differences in Factors Related to HIV Risk Behaviors among People Who Inject Drugs in North-East India

**DOI:** 10.1371/journal.pone.0169482

**Published:** 2017-01-18

**Authors:** Bushra Sabri, Allison M. McFall, Sunil S. Solomon, Aylur K. Srikrishnan, Canjeevaram K. Vasudevan, Santhanam Anand, David D. Celentano, Shruti H. Mehta, Suresh Kumar, Gregory M. Lucas

**Affiliations:** 1 Department of Community and Public Health Nursing, School of Nursing, Johns Hopkins University, Baltimore, Maryland, United States of America; 2 Department of Epidemiology, Bloomberg School of Public Health, Johns Hopkins University, Baltimore, Maryland, United States of America; 3 Department of Medicine, School of Medicine, Johns Hopkins University, Baltimore, Maryland, United States of America; 4 Y.R. Gaitonde Centre for AIDS Research and Education, Chennai, India; Vanderbilt University, UNITED STATES

## Abstract

People who inject drugs (PWID) in India are at high risk for HIV, with women being at elevated risk. Using a socio-ecological framework, this study assessed whether factors associated with HIV transmission risk behaviors differed across men and women PWID. Data for this cross-sectional study were collected from 6449 PWID in 7 cities in Northeast India. Men (n = 5653) and women (n = 796) PWID were recruited using respondent-driven sampling (RDS). We assessed sex differences in two recent HIV transmission risk behaviors: multiple sex partners and needle/syringe sharing. We used multi-level logistic regression models, which incorporated sampling weights and random intercepts for city, to assess factors associated with these HIV risks, separately among men and women. The prevalence of HIV was significantly higher among women than men (53% vs 18.4%, p<0.01). Nearly 13% of men and 8% of women (*p* = .30) had multiple partners. Employment in men and relationship status and stigma in women were significantly associated with multiple partners. Approximately 25% of men and 19% of women engaged in needle sharing (*p* = .16). Younger age in women and depression symptoms in men were significantly associated with increased risk for sharing needles. We found that sexual and drug related risk behaviors were common among PWID in Northeast India, and there were differences between men and women in the socio-ecologic correlates of these behaviors. Contextually-integrated and gender-specific HIV prevention and intervention efforts are needed that consider factors at individual, interpersonal- and community-levels that uniquely impact HIV risks among PWID.

## Introduction

Injection drug use is an important driver of HIV epidemics in different regions of the world [1–3] including India, which is home to 168,000–1.1 million [4, 5] people who inject drugs (PWID) with a high prevalence of HIV and associated risk behaviors[6]. Injection drug use is long endemic in north-east region, putatively due to proximity to the “golden triangle” of heroin production. In contrast to other areas of India, injection drug use is the major driver of the HIV epidemic in the north-east, and high prevalence of HIV have been reported among PWID there [7, 8], with prevalence rates in some cities ranging from 11.2 (Gangtok) to 44.9 percent(Moreh) [8]. This could be attributed to different patterns of drug use[9] with heroin being the most commonly used drug in high HIV prevalence cities (e.g., Imphal, Moreh, Aizawl) compared with low prevalence areas (e.g., Gangtok) in which the most commonly used drugs were reported to be sedatives/antianxiety and other pharmaceutical opioids[8]. The type of drug injected influences how often PWID need to inject and whether the drug can be sometimes taken orally (e.g.,Spasmo-proxyvon) since for some drugs the person does not always have to inject[9]. This can influence their drug-related HIV risk associated with sharing needles and injection drug use. North-east India is distinguished by longstanding civil insurgency, poverty, and unemployment, with many communities located in geographically difficult-to-reach areas[10]. Behaviors associated with acquiring/transmitting HIV among PWID include sexual (e.g., multiple partners) and drug-related behaviors (e.g., sharing needles) [11]. Prior studies in US (8) and Ukraine [12] have found women PWID to be at an elevated risk for HIV when compared to men PWID, which may be due to factors such as social norms (8) (e.g., community acceptance of men’s use of power over women), forced sex and resulting physiological mechanisms leading to HIV risk [13] and women following men in drug use and needing help with injecting. In an analysis of 14,481 PWID in India, the prevalence of HIV infection was three times higher in women than men, an association that persisted even after adjustment for other correlates of HIV infection [8]. Accordingly, the objective here is to study gender differences in HIV risks among PWID in India to better understand the risk contexts for women vs. men PWID. We compared men and women PWID on engagement in sexual HIV risk behaviors such as having multiple sex partners, condomless sex with a non-primary partner, and engagement in exchange sex, and drug-related HIV risk behaviors such as sharing of needles, and daily injection drug use. Additionally, we examined gender differences in factors associated with multiple sex partners and sharing needles [11], two strong predictors of HIV acquisition and transmission among PWID. Although research in India has examined contextual factors related to HIV risk associated with unprotected sex[14] (a factor associated with sexual HIV risk), gender differences in factors associated with multiple sex partners and sharing needles among PWID in India have not been examined.

HIV prevention among PWID requires understanding of the factors that relate to HIV risks. Bronfrenbrenner’s (1977) ecological model can be used to better understand the range of factors at multiple levels in PWID lives that place them at risk for HIV. For instance, at the individual level, lack of education [15], at interpersonal level- drug use networks, and at community level, stigma [16] or lack of access to services [17] can increase risk for HIV. The individual level includes PWID’s demographic, behavioral or health characteristics that are associated with increased engagement in HIV risk behavior. Individual characteristics (e.g., lack of education or knowledge of HIV risks) and use of drugs as a coping mechanism for poor mental health can increase vulnerability to HIV. The interpersonal level, consists of individuals and groups of individuals (such as intimate partner relationships) with whom the individual interact, and assign subjective meanings to those interactions[18, 19]. A partner’s support or preference for protected sex and safe injection practices are protective factors for HIV. Further, social support from family and social networks can reinforce social norms and behaviors that reduce HIV risks [20, 21]. The community level consists of a link between two or more interactions or settings, but the individual is embedded in only one[22]. Stigma manifested at the community level limit the provision and/or uptake of HIV prevention, treatment, and care services[20]. Our objective was to identify gender differences in effects of factors at different ecological levels on sexual and drug related HIV risk behaviors in men and women PWID, with the goal of informing contextually-integrated and gender-specific prevention or intervention efforts in this population.

## Materials and Methods

We analyzed cross-sectional baseline data from a cluster-randomized trial (ClinicalTrials.gov identifier: NCT01686750). We collected data from PWID in 15 cities using respondent driven sampling (RDS) from January to December 2013. To be eligible, participants had to a) be 18 years of age or older, and b) self-report injection drug use in prior 24 months. Each participant was asked to estimate the size of his or her PWID network (i.e., number of PWID in the city whom the participant had seen in the prior 30 days) and to recruit up to two members from this network. The median (range) site-level network size was 20 (7 to 20). RDS recruitment continued until the desired sample size (1000 per site) was reached. RDS performance characteristics were favorable, including recruitment of full samples at each site from only 2 or 3 “seeds”, a large number of recruitment waves (median 21 waves; range 13 to 26), low homophily for HIV status[8], and short recruitment periods (median 94 days; range 52 to 139). All participants were asked to complete the interviewer-administered survey, provide a blood sample, and were offered pre and post-test counseling for HIV. The survey included questions on socio-demographic characteristics and health behaviors. In the present study, analyses were restricted to 7 cities in the northeast (Aizawl, Churachandpur, Dimapur, Gangtok, Imphal, Lunglei, and Moreh) as women PWID are found almost exclusively in this region of India [8]. The study procedures were approved by institutional review boards at YR Gaitonde Centre for AIDS Research and Education (YRGCARE) and the Johns Hopkins Medical School. Participants provided verbal consent since the written consent could compromise participants’ confidentiality and the study procedures were minimal risk. All consent procedures were approved the institutional review boards at YRGCARE and the Johns Hopkins Medical School, Johns Hopkins University

### Measures

#### HIV risk behavior variables

Recent multiple sex partners: Participants reported how many sexual partners they had in the past 6 months. The variable was categorized into one or no partner and two or more partners. Condomless sex with a non-primary partner: Participants were asked how often they used condoms during vaginal and anal sex with their non-primary partners, in the last 6 months. Non-primary partners included casual partner/friend, commercial sex worker, one-time partner or other (excluding spouses and boyfriend/girl-friend). The variable had two categories: Never or sometimes used condom with a non-primary partners and always used condoms with any non-primary partners. Engagement in exchange sex: Participants were asked if they ever had sex to receive money, alcohol, drugs or other things or given money, alcohol or drugs for sex in their lifetime. This variable was categorized as yes or no. Recent sharing of needles/syringes: Participants were asked if they passed a needle to someone else after using it or if they used a needle after someone else had used it. The variable had two categories: participants shared needle in the past 6 months and they never shared a needle. Injected Daily: Participants were asked how often they injected drugs in the last 6 months. This variable was dichotomized to distinguish participants who injected daily from those who injected weekly, monthly or a few times in the last 6 months.

#### Ecological level variables

Among factors at the individual level, age and age at first injection drug use were included as continuous variables. Education was categorized into: primary school or less, secondary school and high school or beyond. Employment categories included employment and unemployment. For financial stress, participants reported how often in the last 12 months they ran out of money for basic necessities, categorized as: a) at least monthly and b) never or less than monthly. For HIV status, participants were categorized according to a testing algorithm that included up to three rapid tests and confirmation by western blot at a central laboratory if indeterminate [8].

Depression was assessed using the Patient Health Questionnaire (PHQ-9; 9 items; alpha = 0.85), a screening measure for assessing depressive symptoms in the past two weeks. The response options ranged from 0 (not at all) to 3 (nearly every day) with total scores ranging from 0 to 27. Higher scores on the PHQ-9 indicate higher levels of depressive symptoms. A score of 10 or higher[23] was used to categorize subjects with clinically significant depression symptoms.

At the interpersonal level of the ecological model, relationship status was measured using two categories: not currently in relationship (i.e., never married, divorced/separated, widowed) and currently in relationship (i.e., currently married or have a partner). Social support was measured using five items (alpha = 0.86) that assessed how often participants had different kinds of support available to them during the past 4 weeks (e.g., help with daily chores). The items were rated using a 4-point scale ranging from 0 (none of the time) to 4 (all of the time), with higher scores indicative of higher levels of social support.

The factor at the community level included enacted stigma. Enacted stigma refers to overt acts of discrimination and hostility directed at individuals [24].The Enacted Stigma Index (6 items; alpha = 0.79) was used to assess how often participants experienced specific discriminatory acts because of their drug use in their lifetime. Participants responded on a 4 point scale (0 = never, 1 = rarely, 2 = sometimes and 3 = frequently). Higher scores indicate more experiences of enacted stigma.

### Data analysis

All variables were compared by gender using descriptive statistics (Tables 1 and 2). Data from RDS ‘seed’ participants were excluded from analyses and a composite weight including the RDS-II (Volz-Heckathorn) weight and the relative population size of PWID in each city derived from state-level data[25] was used to calculate population characteristics. Distributions of characteristics by gender were compared using Pearson chi-squared tests and Wilcoxon rank sum tests for categorical and continuous variables, respectively. Unweighted comparisons are shown in supplemental material in accord with guidelines on reporting RDS results [26]. We used multi-level logistic regression models that include random intercepts for each site (to account for site-to-site variability) and scaled RDS-II weights to explore variables associated with multiple sexual partners and sharing needles/syringes in the past 6 months in separate modeling procedures. First, we ran univariable models for all social-ecological variables separately for men and women. Second, variables with *p* < = 0.1 in univariable models were selected for inclusion in gender-stratified multivariable models, with age included in all models regardless of statistical significance (Tables 3 and 4). Finally, we tested for effect modification by gender on other covariates of interest (age and any factor that was associated *p*< = .10 with a transmission risk behavior in either of the gender-stratified multivariate models) using the following analytical approach: (1) Using the complete cohort, and separately for each outcome (multiple sex partners and needle/syringe sharing), we ran individual models testing the associations of gender, one additional covariate of interest, and interaction term(s) between gender and the additional variable, (2) we constructed separate multivariable models for each outcome in the complete population that included the following explanatory covariates: age, gender, any main effects that were associated (*p*< = 0.1) with the outcome in either sex-stratified multivariate model, and any interaction terms between gender and other covariates that were associated (*p*< = 0.1) in the individual interaction models. In the final multivariate models (gender-stratified or including both genders), *p* values <0.05 were considered statistically significant. Analyses were conducted using Stata Version 12.

**Table 1:** Sample characteristics among men and women who inject drugs in seven cities in India.

	Men % (N)	Women % (N)	P-value
Total (%)	84.1 (5653)	15.9 (796)	
**INDIVIDUAL**			
***Age***, *years* (Mean)	29.7	30.5	0.22
***Age of First Injection Drug Use*** (Mean)	21.0	22.5	0.13
***Education*** (%)			
Primary School or Less	24.7(1062)	29.8 (243)	
Secondary School	53.1 (3023)	56.6 (439)	0.18
High School or Beyond	22.1 (1568)	13.5 (113)	
***Employment*** (%)			
Employed	72.0 (4102)	52.0 (520)	
Unemployed	27.9 (1551)	47.9 (276)	<0.001
***Frequency of Financial Stress*** (i.e. run out of basic necessities) (%)			
Less than monthly	37.4 (2149)	31.7 (260)	
Monthly or more frequently	62.5 (3166)	68.2 (481)	0.27
***Psychological***			
Depressed*	38.2 (1853)	38.3 (306)	0.95
***HIV Status***			
HIV negative	81.6 (4521)	47.0 (427)	
HIV-positive	18.4 (1132)	53.0 (368)	<0.01
**INTERPERSONAL**			
***Relationship***			
Not currently in relationship	50.6 (2970)	44.5 (413)	
Currently in relationship (i.e., married or long-term partner)	49.3 (2682)	55.4 (383)	0.07
***Social Support*** (Mean)	3.43	3.29	0.37
**COMMUNITY**			
***Enacted Stigma*** (Mean)	0.36	0.25	0.13
***North-East Cities***			
Aizawl	15.0 (851)	18.7 (149)	
Churchandpur	14.1 (800)	25.1 (200)	
Dimapur	15.7 888)	14.0 (112)	<0.001
Gangtok	16.7 (948)	6.53 (52)	
Imphal	9.42 (923)	9.67 (77)	
Lunglei	15.6 (884)	14.2 (113)	
Moreh	6.35 (359)	11.6 (93)	

Note: Percentages represent within gender percentages;

*Depression was measured using Patient Health Questionnaire (PHQ-9)

**Table 2:** Gender differences in sexual and drug-related HIV risk behaviors among men and women who inject drugs in seven cities in India (N = 6449).

	Men % (N)	Women % (N)	P-value
**SEXUAL HIV RISK BEHAVIORS**			
Multiple sex partners in the past 6 months TOTAL	12.8 (748)	7.6 (113)	0.30
HIV negative	13.3 (649)	8.6 (63)	0.19
HIV-positive	10.7 (99)	6.7 (50)	0.45
Condomless sex with a non-primary partner in past 6 months TOTAL	49.0 (2699)	46.0 (382)	0.38
HIV negative	51.0 (2307)	53.0 (225)	0.75
HIV-positive	42.0 (392)	41.0 (157)	0.86
Ever engaged in exchange sex TOTAL	22.8 (1326)	21.2 (149)	0.55
HIV negative	23.3 (1095)	18.1 (79)	0.71
HIV-positive	21.1 (231)	24.1 (70)	0.48
**DRUG-RELATED HIV RISK BEHAVIORS**			
Shared needle/syringe in past 6 months TOTAL	24.9 (2131)	18.9 (272)	0.16
HIV negative	25.5 (1794)	26.3 (169)	0.83
HIV-positive	22.3 (337)	12.4 (103)	0.12
Injected Daily in past 6 months TOTAL	65.7 (3623)	66.4 (447)	0.83
HIV negative	65.3 (2974)	58.9 (250)	0.16
HIV-positive	67.8 (649)	75.9 (196)	0.10

Note: Percentages represent within gender percentages

**Table 3:** Factors associated with multiple recent sex partners in men and women who injected drugs in seven cities in India (N = 6449).

	Univariable analysis		Multivariable analysis	
Factor	Women	Men	Women	Men
	OR (95% CI)	OR (95% CI)	OR (95% CI)	OR (95% CI)
**INDIVIDUAL**				
***Age***	0.86 (0.58–1.26)	0.93 (0.82–1.05)	0.83 (0.57–1.20)	0.91 (0.77–1.07)
***Age of first injection drug use***	0.95 (0.67–1.33)	0.88(0.78–0.99)*	-	0.91 (0.76–1.09)
***Education***				
High School/Beyond	1.09 (0.36–3.37)	0.63(0.39–1.02)	-	0.72 (0.46–1.12)
Secondary	1.82 (0.83–3.97)	0.86(0.68–1.07)	-	0.91 (0.77–1.08)
Primary School /Less (Ref)	1	1		1
***HIV status***				
HIV Positive	1.55 (0.47–5.16)	1.01 (0.58–1.78)	-	-
HIV negative (Ref)	1	1		
***Employment***				
Employed	0.92 (0.43–1.99)	1.28 (1.03–1.59)*	-	1.35 (1.06–1.72)*
Unemployed (Ref)	1	1		1
***Financial Stress***				
Monthly/more frequently	1.05 (0.58–1.89)	1.25 (0.83–1.86)	-	-
Less than monthly (Ref)	1	1		
***Psychological***				
Depression	2.09 (1.02–4.28)*	1.44 (0.94–2.21)	2.19 (0.97–4.90)	1.46 (0.99–2.16)
**INTERPERSONAL**				
***Relationship***				
Currently in relationship	0.36 (0.23–0.58)***	1.08 (0.78–1.48)	0.33 (0.18–0.62)**	1.09 (0.76–1.59)
Not in relationship (Ref)	1	1	1	1
***Social Support***	0.82 (0.55–1.22)	0.94 (0.81–1.08)	-	-
**COMMUNITY**				
***Enacted Stigma***	1.16 (1.07–1.26)***	1.03 (0.96–1.11)	1.16 (1.08–1.48)***	-

*p < .05;

**p < .01;

***p < .001

**Table 4:** Factors associated with recent needle sharing in men and women who injected drugs in seven cities in India (N = 6449).

	Univariate analysis		Multivariate analysis	
Factor	Women	Men	Women	Men
	OR (95% CI)	OR (95% CI)	OR (95% CI)	OR (95% CI)
**INDIVIDUAL**				
***Age***	0.81 (0.69–0.94)**	0.83 (0.74–0.93)**	0.78 (0.65–0.94)**	0.89 (0.77–1.01)
***Age of first injection drug use***	0.88 (0.67–1.14)	0.81 (0.65–0.99)*	-	0.89 (0.75–1.06)
***Education***				
High School/Beyond	1.09 (0.36–3.37)	0.53 (0.29–0.96)*	-	0.49 (0.28–0.88)*
Secondary	1.82 (0.83–3.97)	0.71 (0.52–0.97)*	-	0.68 (0.48–0.96)*
Primary School /Less (Ref)	1	1		1
***HIV status***				
HIV Positive	0.69 (0.40–1.20)	0.69 (0.48–1.01)	-	0.79 (0.56–1.11)
HIV negative (Ref)	1	1		1
***Employment***				
Employed	0.73 (0.43–1.24)	0.82 (0.60–1.11)	-	-
Unemployed (Ref)	1	1		
***Financial Stress***				
Monthly/more frequently	1.14 (0.83–1.57)	1.34 (0.79–2.26)	-	-
Less than monthly (Ref)	1	1		
***Psychological***				
Depression	0.99 (0.67–1.48)	1.36(1.13–1.64)**	0.98 (0.71–1.34)	1.42 (1.14–1.75)**
**INTERPERSONAL**				
***Relationship***				
Currently in relationship	1.23 (0.86–1.75)	0.63 (0.49–0.79)***	-	0.68 (0.51–0.91)**
Not in relationship (Ref)	1	1		1
***Social Support***	0.75 (0.46–1.21)	0.77 (0.54–1.09)	-	-
**COMMUNITY**				
***Enacted Stigma***	1.06 (1.02–1.11)**	1.05 (0.99–1.12)	1.07 (1.02–1.12)**	-

*p < .05;

**p < .01;

***p < .001

## Results

### Participant characteristics

The sample consisted of 6449 PWID from 7 cities in northeast India; 5653 men (84.1%) and 796 women (15.9%, Table 1). About 57% of women and 53% of men had secondary education (*p* = 0.18). Nearly half (47.9%) of women and a quarter of men (27.9%) were unemployed (*p*<0.001). More than 60% of men and women reported frequent experiences of financial stress. Although not statistically significant, more men than women reported not being in a current relationship (*p* = 0.07).

More than half of the women were HIV positive (53.0% vs 18.4% men; *p* < .01). Around 38% of both men and women reported clinically significant depression symptoms. Men reported higher levels of enacted stigma than women (mean = 0.36 vs 0.25 women; *p* = 0.13), although the difference was not statistically significant.

### Gender differences in HIV risk behaviors

There were no statistically significant differences in sexual and drug related behaviors by gender (Table 2). Although gender differences in risk behaviors were not statistically significant, more men than women reported having recent multiple sex partners (12.8% vs 7.6% women; *p* = 0.30), a trend that held in subsets stratified by HIV status. Overall, more men than women reported condomless sex with a non-primary partner (*p* = 0.38) or engagement in exchange sex (*p* = 0.55). Similarly, men were more likely to report recent sharing of needles (24.9% vs 18.9% women, *p* = 0.16). Overall, HIV positive individuals were less likely to report transmission risk behaviors than HIV negative individuals (except for daily injection among both men and women and for exchange sex among women). The gender difference was most marked among HIV positive women for needle sharing (12.4%, *n* = 103 among HIV positive women versus 22.3%, *n* = 337 among HIV positive men; *p* = 0.12; Table 2).

### Gender differences in factors associated with HIV risk behaviors

#### Recent multiple sex partners

Women (Table 3), in relationships were significantly less likely than those who were not in relationships, to report multiple partners (*AOR* = 0.33, *95% CI* = 0.18–0.62; *p*<0.01). However, there was no association between relationship status and multiple partners among men. This effect modification by gender on the association between relationship status and multiple partners persisted in the final multivariate interaction model (Women: *AOR* = 0.32, *95% CI* = 0.16–0.64; Men: *AOR* = 1.10, *95% CI* = 0.81–1.49; *p*<0.01 for interaction). Among men, employment was significantly related to multiple recent sex partners *AOR* = 1.35, *95% CI* = 1.06–1.72; *p*<0.05). Stigma (*AOR* = 1.16, *95% CI* = 1.08–1.48; *p*<0.001) was positively associated with having multiple partners among women, but not among men. In the final model, gender differences in association of stigma with multiple partners were supported by significant interaction between gender and stigma (Women: *AOR* = 1.17, *95% CI* = 1.08–1.27; Men: *AOR* = 1.03, *95% CI* = 0.96–1.09; *p* < .01 for interaction).

#### Recent sharing of needles/syringes

Among women, younger age (*AOR* = 0.78 per 5 years, *95% CI* = 0.65–0.94; *p*<0.01) and enacted stigma (*AOR* = 1.07, *95% CI* = 1.02–1.12; *p*<0.01) were significantly associated with greater odds of needle sharing. Education was a significant factor associated with needle sharing among men. Among men, needle sharing was less common among those with secondary education (*AOR* = 0.68, *95% CI* = 0.48–0.96; *p*<0.05) or with high school education or above (*AOR* = 0.49, *95% CI* = 0.28–0.88; *p*<0.05) (Table 4). Further, compared to those who were not in relationships, being in relationships (*AOR* = 0.68, *95% CI* = 0.51–0.91; *p*<0.01) was associated with lower likelihood of needle sharing among men. Depression (*AOR* = 1.42, *95% CI* = 1.14–1.75; *p*<0.01) significantly increased likelihood of needle sharing in men. In the interaction model, we found that the interaction between gender and education was the only statistically significant interaction (*p*<0.05), indicating that higher education may reduce drug-related HIV risk among men (secondary education (*AOR* = 0.71, *95% CI* = 0.51–0.98; high school education or above, *AOR* = 0.52, *95% CI* = 0.29–0.93) but not among women.

## Discussion

In this study, we found high prevalence of HIV and recent transmission risk behaviors, among both men and women PWID from north-east India. Despite the higher HIV prevalence among women as compared to men, we found no statistically significant differences in the prevalence of HIV risk behaviors between men and women. Moreover, we observed that socio-ecologic correlates of recent HIV transmission risk behaviors differed in men and women.

At the individual level of the ecological model, employment status was significantly associated with sexual HIV risk of recent multiple sex partners among men while this relationship was not significant among women. This could be explained by employed men having more resources to engage in multiple sexual relationships than unemployed men. Younger age was independently associated with increased needle sharing among women while among men this association was non-significant after adjustment for other factors. Prior research shows that younger PWID may have lower perception of HIV risk and inadequate HIV prevention education [27]. Other research shows that drug use initiation at an early age was related to the first sexual experience at a younger age, and to subsequent HIV risk behaviors such as multiple sex partners [28]. Thus enhanced prevention efforts are needed to reduce HIV risk among women in the younger age group.

Other individual level factors such as low education and depression were related to needle sharing among men. By contrast, for women needle sharing was only associated with younger age. The association between lower education and sharing needles among men could be attributed to poor access to HIV education, and inability to understand HIV risk messages [29]. By contrast, education had no association with HIV risk among women. Since women often have limited decision-making power in Indian culture, other factors (e.g., risk posed by their partner’s risky behaviors) may play a more significant role in women’s HIV risks than their own level of education. Regardless, programs are needed to educate PWIDs on HIV/AIDs and behaviors that place them at risk of contracting HIV. Further, depression was significantly related to drug-related HIV risk among men. The findings are similar to a previous study in India in which PWID with severe depression symptoms were more likely to share needles/syringes [30]. Depressed PWID may engage in HIV risk behaviors to mitigate distress [31, 32]. Depression has been associated with carelessness and poor social and negotiation skills when navigating risky scenarios with peer injectors, which explains association of depression with needle sharing[30]. Depression has also been linked to reduction in libido and sexual activity[30, 33] that may explain the absence of an association between depression and sexual risk behaviors. Thus, efforts are needed to assess for depression and its impact on HIV risk among men, with increased access to mental health counseling services for at-risk PWID.

The interpersonal level of the ecological model (i.e., having an intimate partner versus not) determined risk for HIV among PWID. Research in other countries shows that HIV risks are highly influenced by relationship context due to behavioral norms [34]. Being in a relationship was associated with lower likelihood of multiple sex partners among women. In contrast, men who were in relationships were similarly likely to report multiple partners as men who were not in relationships. In India, women who are single, in particular, may be socially, economically and sexually vulnerable placing them at greater risk for risky behaviors such as sharing needles [35]. Thus, while being in a current relationship was found to be protective against women’s engagement in HIV sexual risk behavior of multiple sexual relationships, being in a current relationship was protective against men’s injection risk behaviors (i.e., needle sharing).

At the community level of the social ecological model, we found significant relationships between stigma and HIV risk behaviors among PWID, as supported by prior research in India [36]. Compared to women, men reported significantly higher mean levels of enacted stigma. This could be attributed to men seeking more services and experiencing stigma in their communities. Although stigma was reported significantly more frequently by men than women, stigma was strongly correlated with multiple partners and sharing needles in women, but not in men. It is likely that women who engage in multiple relationships and also use drugs experience stigma due to gender norms about appropriate behaviors for women (e.g., being caregivers of the family).

Research shows that illicit drug use is more stigmatized than other chronic medical or psychiatric conditions, perhaps because PWID are perceived as having control over the drug use, and are thus more likely to be blamed for their problems [36]. PWID excluded from their broader social network may turn to drugs using social network for support and continue their risky behaviors [37]. Experiences of discrimination (e.g., denial of care), inability to quit and resulting psychological distress[24] may expose PWID to HIV risk. Stigma may prevent PWID from seeking treatment and may lead to increased involvement in HIV risk behaviors [38]. Therefore, there is need for HIV prevention/risk reduction approaches that address stigma, reduce discrimination and increase access to services among PWID.

A social-ecological framework can be used to identify factors associated with HIV risks among PWID. It is important to consider roles of factors at individual, interpersonal and community levels (e.g., depression and mistreatment due to stigma) in risk for HIV. Further, it is important to examine gender differences in impact of factors on HIV risk. Similar to previous research in the US (30), our study suggests that men and women PWID in India have different prevention needs and risk factors associated with HIV risk. Efforts to prevent the transmission of HIV among PWID must incorporate gender-focused approaches, including addressing gender norms that contribute to HIV risks. In our study, a greater proportion of women than men were found to be HIV positive. Women can be at increased risk for HIV due to factors such as forced sex [39]. In Indian context, inability to negotiate safe sex practices with partners may place women at risk for HIV. Moreover, women’s access to drugs and other resources are often controlled by their male partners[40]. Women need help with injection leading to needle sharing, are less comfortable injecting in public and face increased difficulty in accessing services [40]. Educational and awareness programs are needed that focus actively on harm reduction and prevention of HIV transmission risks among HIV-infected women PWID and risks of HIV-infection among at-risk women PWID.

In a study in North-east India, drug using women expressed the needs of women-only integrated health, detoxification and rehabilitation services, mental health services and assistance to meet basic needs [35]. This shows that gender should be an important consideration in HIV prevention and intervention services for PWID and in addressing the individual, community and institutional impacts on their HIV risks.

Our study strengths include a large sample size and systematic sampling methodology with analytic weighting of estimates. Limitations include self-report measures, with the exception of HIV status. Further, we did not collect data on other potential factors that may underlie sex differences in HIV risk among PWID in India such as forced sex and access to drugs and injection paraphernalia. In addition, the study was cross-sectional and we were unable to determine the direction of causality. Despite these limitations, the study provides findings on differences between men and women in the socio-ecologic correlates of HIV risks, which support the need for contextually integrated and gender specific HIV prevention and interventions for PWID.
